# A Prototypical Small-Molecule Modulator Uncouples Mitochondria in Response to Endogenous Hydrogen Peroxide Production

**DOI:** 10.1002/cbic.201300115

**Published:** 2013-05-02

**Authors:** Stephen J McQuaker, Casey L Quinlan, Stuart T Caldwell, Martin D Brand, Richard C Hartley

**Affiliations:** [a] WestChem School of Chemistry, University of GlasgowGlasgow, G12 8QQ (UK) E-mail: Richard.Hartley@glasgow.ac.uk; [b] Buck Institute for Research on Aging8001 Redwood Boulevard, Novato, California 94945 (USA)

**Keywords:** antioxidants, bioorganic chemistry, drug delivery, mitochondria, prodrugs

## Abstract

A high membrane potential across the mitochondrial inner membrane leads to the production of the reactive oxygen species (ROS) implicated in aging and age-related diseases. A prototypical drug for the correction of this type of mitochondrial dysfunction is presented. MitoDNP-SUM accumulates in mitochondria in response to the membrane potential due to its mitochondria-targeting alkyltriphenylphosphonium (TPP) cation and is uncaged by endogenous hydrogen peroxide to release the mitochondrial uncoupler, 2,4-dinitrophenol (DNP). DNP is known to reduce the high membrane potential responsible for the production of ROS. The approach potentially represents a general method for the delivery of drugs to the mitochondrial matrix through mitochondria targeting and H_2_O_2_-induced uncaging.

*In memory of Guy Dodson*.

## Introduction

The average age of the world's population is steadily increasing, and so it is important to individuals, societies and the global economy that the burden of age-related disease is reduced and healthspan (the proportion of a person's life for which he or she is healthy and active) is maximised.^1^ Oxidative stress is involved in almost all diseases in which age is the primary risk factor, including cardiovascular disease, neurodegeneration and stroke, and is implicated in the process of ageing itself.^2^ There is evidence that mitochondria are the main endogenous source of the reactive oxygen species (ROS) that give rise to this oxidative stress,^3^ producing the first ROS, superoxide (O_2_^⋅−^), as a side-product of oxidative phosphorylation through incomplete reduction of molecular oxygen by electrons leaked from complexes I, II and III of the electron-transport chain (ETC).^4^ Superoxide dismutase within the mitochondria catalyses the rapid dismutation (disproportionation) of superoxide to give oxygen and hydrogen peroxide. When H_2_O_2_ interacts with redox-active metal ions [e.g., copper(I) and iron(II)], it produces highly reactive hydroxyl radicals by the Fenton reaction, and these damage biomolecules. This ROS-mediated damage can occur in the mitochondria themselves, but also elsewhere, as H_2_O_2_ can readily diffuse across mitochondrial and cellular membranes. The body's antioxidant defence and repair mechanisms generally keep ROS-mediated damage low, and H_2_O_2_ is an important signalling molecule for maintaining homeostasis, but oxidative damage increases with increasing age and does so dramatically in a wide range of age-related pathologies. Indeed, a vicious cycle of oxidative damage to mitochondrial DNA (mtDNA) resulting in a defective ETC, which in turn gives higher ROS production,^3^ has been proposed as a mechanism of aging in some versions of the mitochondrial free-radical theory of aging.^5^

**Figure 1:**
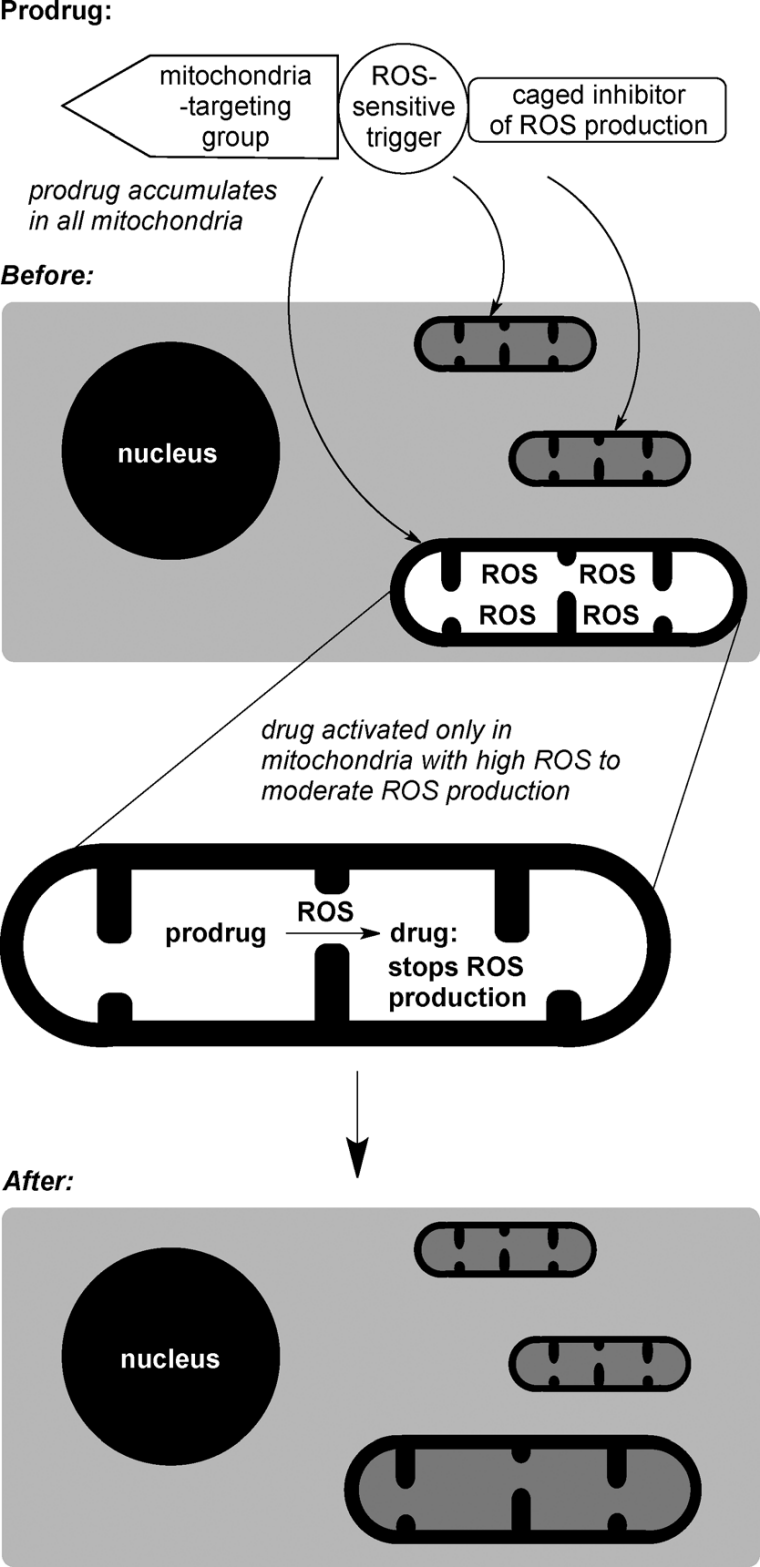
Prodrugs to correct dysfunctional mitochondria. The prodrug accummulates in all mitochondria because of its targeting group, but is only activated in mitochondria where ROS levels are high, with uncaging triggered by a ROS-sensitive group; the drug itself then interferes with the process that is giving rise to ROS, so that normal ROS levels are restored within the dysfunctional mitochondria.

Most cells have many mitochondria, and each of these mitochondria can have several copies of mtDNA. As a result, even within a single cell, it is unlikely that mitochondrial damage and dysfunction is homogeneous; instead, some mitochondria function well, whereas others function poorly, and may be preferentially cleared by mitophagy. We posed the question: is it possible to modulate ROS production in the “misbehaving” mitochondria without adversely affecting overall ATP production or important ROS-dependent processes such as intracellular signalling^6^ and immune responses?^7^ Conceptually, we require functional molecules, which could be regarded as prodrugs, that accumulate in all mitochondria as a result of a targeting group, but are activated through a ROS-dependent trigger only in those mitochondria where ROS levels are high enough to release molecules that moderate ROS production (Figure 1). Thus, an instruction issued by an individual “misbehaving” mitochondrion (i.e., one producing high levels of ROS) would induce a localised response. This strategy would complement our recently reported externally instructed, mitochondria-targeted functional molecules, which release an active compound within an individual mitochondrion with spatial and temporal control in response to irradiation by a laser.^8^

## Results and Discussion

**Figure 2:**
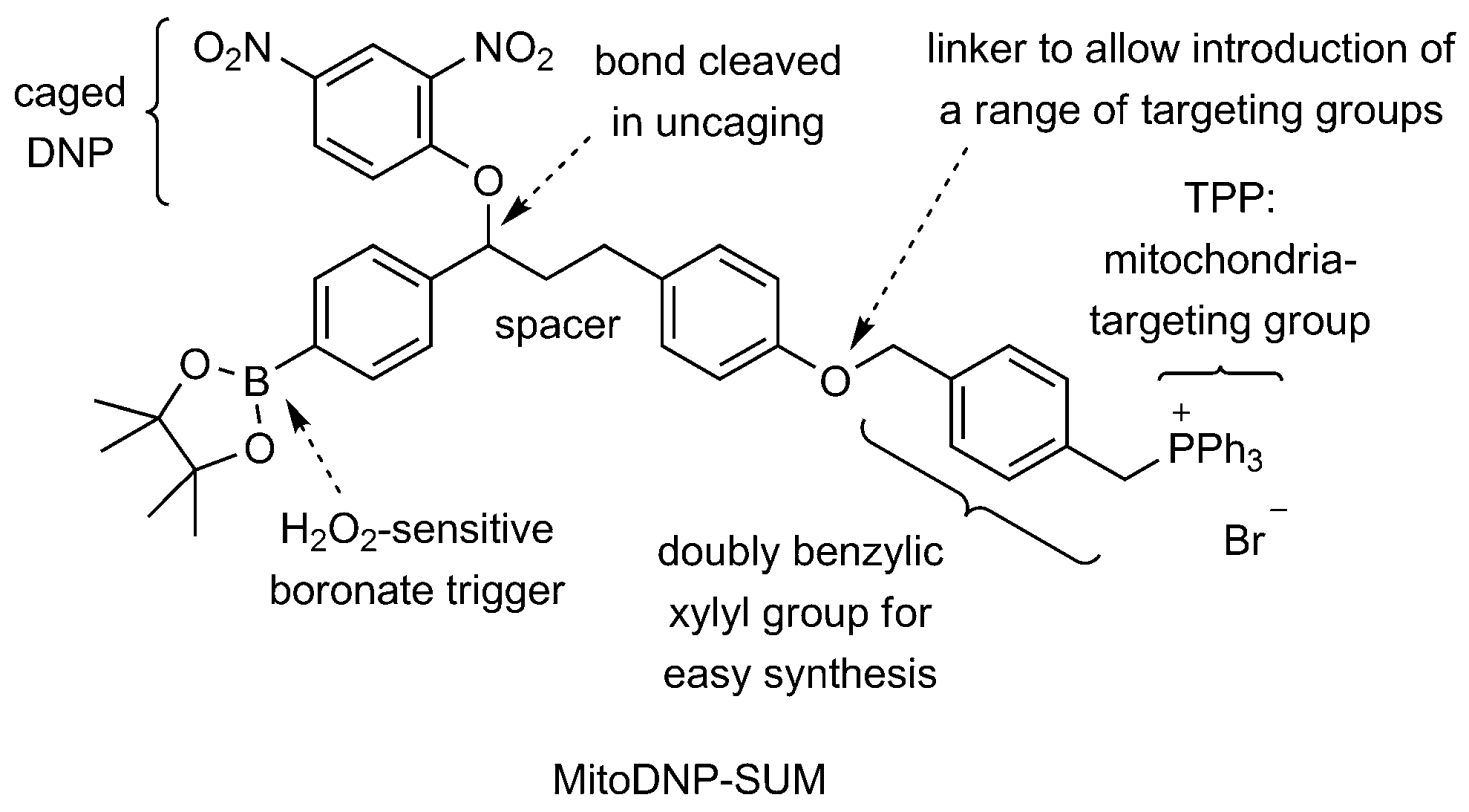
Design features of MitoDNP-SUM.

The design of our prototypical molecule arises from the biological events at the mitochondrial inner membrane (MIM). The ETC pumps protons out of the mitochondrial matrix into the intermembrane space, and this leads to a proton motive force (Δ*p*) composed of a proton concentration gradient (ΔpH, alkaline inside) and a membrane potential (Δ*ψ* negative inside) across the MIM. This drives the flow of protons back into the matrix through ATP synthase powering ATP synthesis. If Δ*p* rises sufficiently, electron transport stalls; thus leading to high reduction of electron carriers in the chain, and reduction of oxygen to superoxide at complexes I, II and III;^4^ this can overwhelm the antioxidant defences. However, even a small reduction in Δ*p* is enough to greatly decrease ROS production, particularly that caused by reversed electron tranport into complex I.^9^ Our prototypical small-molecule modulator or selective uncoupling molecule (SUM), MitoDNP-SUM (Figure 2), was designed to accumulate in the mitochondrial matrix, and respond to H_2_O_2_ specifically in those mitochondria where the ROS levels are high and respond by releasing a proton translocator (an uncoupler) that would transport protons across the MIM, thus reducing Δ*p* (uncoupling electron tranport from ATP synthase) and attenuating ROS production. MitoDNP-SUM could be considered to be a small-molecule mimic of the mitochondrial proteins UCP2 and UCP3, which can cause mild uncoupling in response to oxidative stress.^10^

MitoDNP-SUM (Figure 2) has three important functional features: an alkyltriphenylphosphonium (TPP) group to target the prodrug to the mitochondrial matrix; an arylboronate group to act as a H_2_O_2_-sensitive trigger; and a 2,4-dinitrophenoxyl group that is a caged form of the classic uncoupler, 2,4-dinitrophenol (DNP).

Lipophilic cations^11^ and mitochondria-penetrating peptides^12^ are the best established methods of targeting small molecules to the mitochondrion. The TPP group is cationic and sufficiently lipophilic to diffuse across biological membranes, so TPP compounds accumulate several hundred-fold in the mitochondrial matrix as a result of the mitochondrial membrane potential (Δ*ψ*) in accordance with the Nernst equation.^11^ Tests on MitoQ (TPP attached to a ubiquinone moiety) show that the TPP group does not adversely affect pharmacokinetics or induce toxicity, as this TPP compound can be delivered effectively by a variety of means, including orally, and without toxicity.^11^

The reaction of arylboronates with H_2_O_2_ to give phenols is the basis of a fluorescent probe^13^ and a mass spectrometric probe^14^ for the measurement of H_2_O_2_ levels in the mitochondria of cells and whole organisms, respectively. Another ROS, peroxynitrite also induces this reaction,^15^ but other biological molecules do not convert arylboronates to phenols. MitoDNP-SUM uses this reaction to induce fragmentation and release an uncoupler, a trigger mechanism that was first demonstrated for the H_2_O_2_-induced release of fluorophores.^16^ DNP was chosen as the caged uncoupler; it is the classic example of an uncoupler,^17^ being a weak acid (p*K*_a_ 4.1^18^) and able to cross membranes in both its protonated and deprotonated forms.^19^ Once released, DNP shuttles protons across the MIM by diffusing into the mitochondrial matrix, releasing a proton and then diffusing out again as the corresponding phenoxide (DNP^−^) to collect another proton. The uncoupler molecule is caged in MitoDNP-SUM by attachment through its phenolic oxygen atom to prevent uncoupling occuring prior to H_2_O_2_-triggered release.

**Scheme 1:**
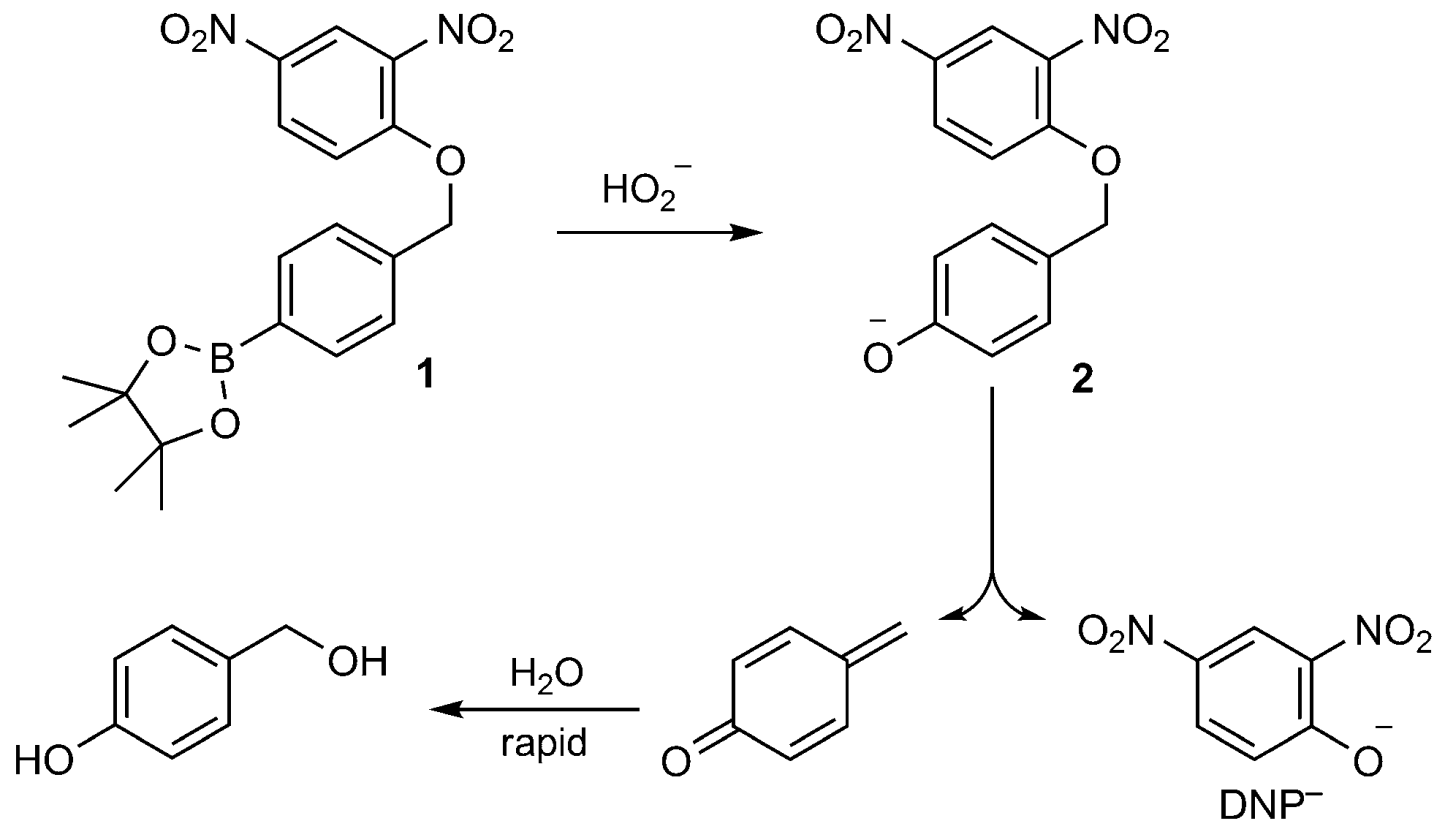
H_2_O_2_ reacts with an arylboronate cage to release DNP.

We have previously shown that the conversion of arylboronate **1**, which is related to phenoxide **2**, leads to the release of DNP with a second-order rate constant of (10±0.8) m^−1^ s^−1^ (Scheme 1).^20^ Release of the uncoupler by this mechanism will continue as long as there is high ROS production in the “misbehaving” mitochondrion, so the SUM should be effective even though some uncoupler will diffuse away. In accordance with the second-order rate equation, the rate of release will drop in proportion to the falling [H_2_O_2_]. As a small reduction in Δ*p* is known to attenuate H_2_O_2_ production dramatically,^9^ uncoupler release will be abruptly reduced to low levels as soon as the [H_2_O_2_] in the mitochondrion drops. This means that the selectivity for “misbehaving” mitochondria should be high, with most mitochondria little affected by the SUM because their [H_2_O_2_] will be low (the level in humans is not known, but 177 nm is a good estimate for average mitochondrial [H_2_O_2_] in young *Drosophila*^14^). The targeted nature of MitoDNP-SUM is also important in preventing off-target effects. Its concentration would be expected to be about 300 times higher in the mitochondrion than in the cytosol. Reaction will also be faster in the mitochondrion because the pH of the matrix is about 0.8 pH units higher than that of the cytosol, and the conjugate base of H_2_O_2_ is the reactive species (Scheme 1).^14^ We have previously demonstrated that the localised uncoupling of selected mitochondria can be achieved by releasing DNP into the matrix of these mitochondria through photo-uncaging of a mitochondrion-targeted precursor.^8^

A two-carbon spacer group is positioned in MitoDNP-SUM to ensure that nonspecific elimination of DNP is not encouraged by conjugation between the trigger and the central aromatic group. Synthetic considerations also played a part in the design of MitoDNP-SUM. The use of an aryl ether link meant that the targeting group would be introduced last and so could potentially be varied, and the xylyl group provided two benzylic sites for S_N_2 reactions.

An earlier compound, MitoDNP (not shown), combines a TPP-targeting group and dinitrophenol,^21^ but differs from MitoDNP-SUM (trigger and caged DNP shown) in two critical ways: the dinitrophenol group is not caged, so it would affect all mitochondria regardless of ROS production, and the dinitrophenol unit is permanently attached to the lipophilic cation. The latter feature might explain the lack of efficacy of MitoDNP, as efflux of the deprotonated MitoDNP^−^ from the matrix, which would be necessary for proton translocation, would be disfavoured by TPP. Here we demonstrate the unique responsive nature of MitoDNP-SUM: it reacts with H_2_O_2_ made by mitochondria to release enough DNP to give significant levels of uncoupling.

**Scheme 2:**
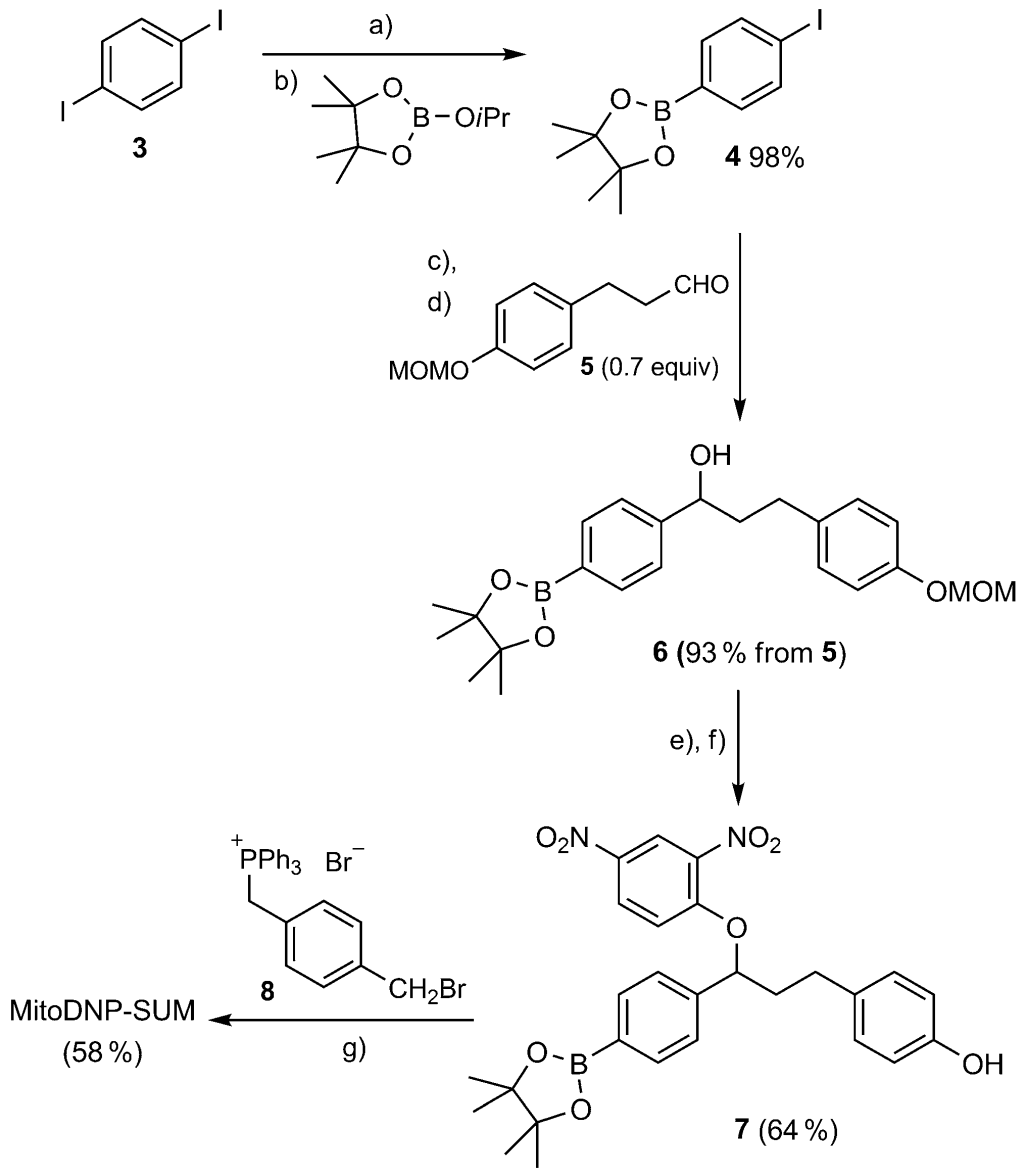
Synthesis of MitoDNP-SUM. a) *i*PrMgCl, LiCl, THF, −78 °C; b) −78 °C to RT; c) THF, LiCl, *i*PrMgCl, −78 °C; d) −78 °C to RT; e) 1-fluoro-2,4-dinitrobenzene, Et_3_N, acetone, 40 °C; f) HCl, MeOH/CH_2_Cl_2_, RT; g) Cs_2_CO_3_, MeCN, RT.

MitoDNP-SUM was synthesised as shown in Scheme 2. The arylboronate trigger of MitoDNP-SUM was installed by using Baron and Knochel's chemoselective magnesiation of aryl iodides.^22^ First, monomagnesiation of 1,4-diiodobenzene (**3**) followed by borylation gave arylboronate **4**, then magnesiation and reaction with aldehyde **5** gave alcohol **6**. Nucleophilic aromatic substitution of the fluoride of 1-fluoro-2,4-dinitrobenzene, followed by deprotection gave phenol **7**, which incorporates the caged DNP and could potentially be attached to any targeting group. This was then alkylated with TPP-bearing benzylic bromide **8**, easily prepared from α,α′-*para*-dibromoxylene and triphenylphosphine, to give the target compound.

**Figure 3:**
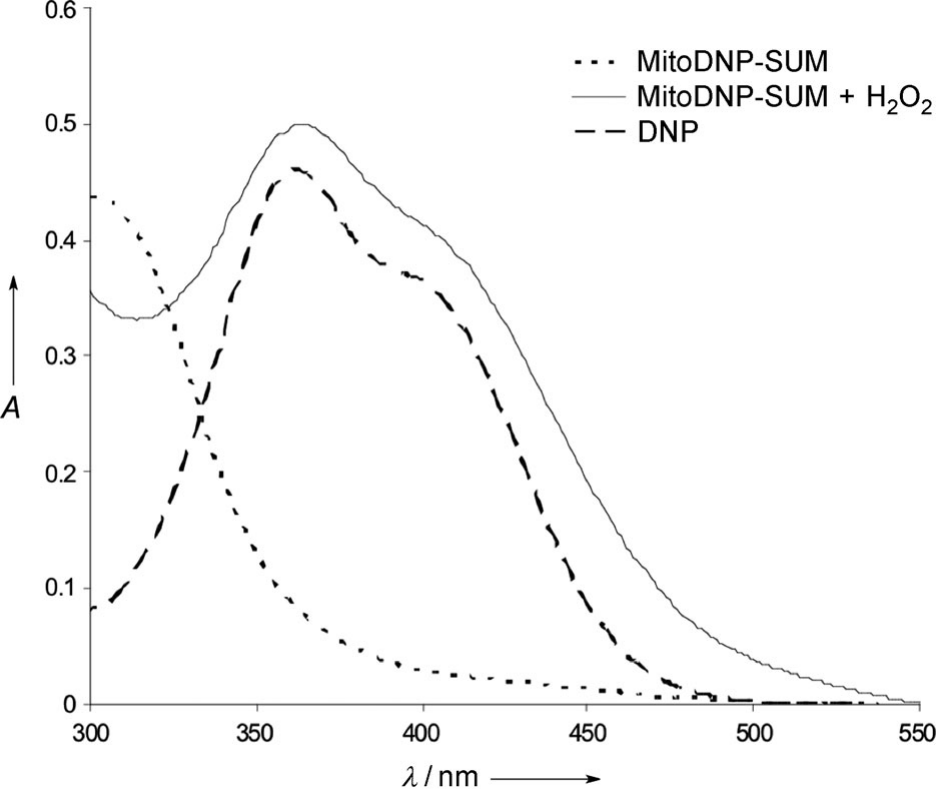
UV–visible spectra illustrating the H_2_O_2_ sensitivity of MitoDNP-SUM in vitro. The H_2_O_2_-induced release of DNP was confirmed by comparing the UV–visible spectra of 41.4 μm MitoDNP-SUM at 37 °C in aqueous KHE buffer (2 % acetonitrile) adjusted to pH 8.0 (the approximate pH of the mitochondrial matrix) with the same concentration of MitoDNP-SUM treated with 4 mm H_2_O_2_ for 15 min and with 41.4 μm of DNP in the same buffer system. All experiments were carried out in triplicate, and the average is presented.

The design principles of MitoDNP-SUM are ideally tested in isolated mitochondria. MitoDNP-SUM should accumulate in energised mitochondria and release free DNP as a result of high endogenous H_2_O_2_ production. In the test tube, we found that free DNP is released from MitoDNP-SUM after exposure to H_2_O_2_ (Figure 3).

**Figure 4:**
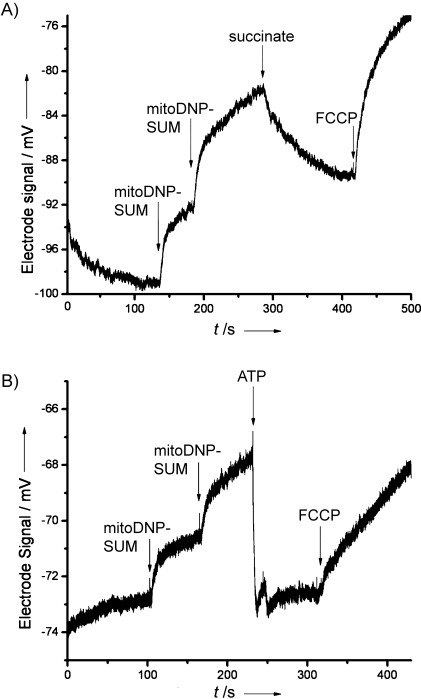
An electrode sensitive to the TPP moiety of MitoDNP-SUM was conditioned in KHE buffer and 10 μm methyltriphenylphosphonium (TPMP) for two weeks prior to the experiment, then washed extensively before use to remove TPMP. Rat skeletal muscle mitochondria (0.3 mg protein per mL) were incubated in KHE plus 0.3 % (*w*/*v*) BSA with stirring at 37 °C. A) Two aliquots of 3 μm MitoDNP-SUM were added to the chamber, and mitochondrial uptake was initiated by the addition of succinate (5 mm). Release of MitoDNP-SUM was instigated by the addition of FCCP (4 μm). B) Mitochondria were incubated in the presence of antimycin A (2 μm). Two aliquots of 3 μm MitoDNP-SUM were added to the chamber, and the mitochondria were energised by hydrolysis of ATP (5 mm). Release was initiated by FCCP.

In isolated mitochondria, the uptake of MitoDNP-SUM was monitored with an electrode sensitive to the TPP moiety. Mitochondria incubated in the presence of rotenone, an inhibitor of complex I of the ETC, do not establish a membrane potential and do not take up MitoDNP-SUM (Figure 4 A), but upon addition of succinate, which is a substrate of complex II, the inhibition is bypassed, and Δ*ψ* drives uptake of the compound as expected. The collapse of Δ*ψ* induced by the uncoupler carbonyl cyanide 4-(trifluoromethoxy)phenylhydrazone (FCCP) causes a release of the compound. Antimycin A inhibits complex III of the ETC and prevents the generation of Δ*ψ* in a way that cannot be bypassed by these substrates of the ETC (Figure 4 B). However, Δ*ψ* can be generated by driving ATP synthase in reverse through ATP hydrolysis, and this again leads to the uptake of MitoDNP-SUM but in an ETC-independent manner.

**Figure 5:**
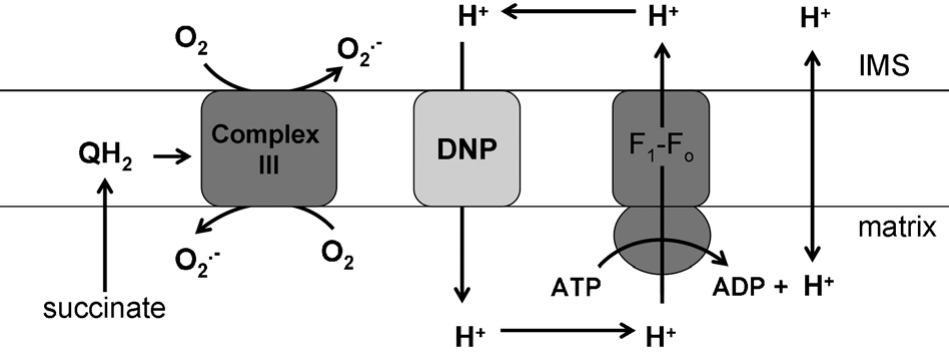
Experiment designed to test ROS-dependent uncoupling. A constant membrane potential was generated by ATP hydrolysis. During ATP hydrolysis, in addition to vectorial proton pumping, scalar protons are liberated, and this results in net acid production and a corresponding drop in the pH of the medium. In the basal state, Δ*p* maintained by ATP hydrolysis is high because of limited proton conductance, therefore ATP hydrolysis and the net acidification of the medium are slow. The addition of an uncoupler (DNP) will result in an increased rate of ATP hydrolysis because of increased proton conductance. Under high-ROS conditions, greater uncoupling is achieved because more DNP is released from the MitoDNP-SUM. High-ROS conditions were generated by incubating mitochondria with succinate (5 mm) and antimycin A (2 μm), this led to superoxide production from complex III.^23^ Low-ROS conditions were created by omitting the succinate.

**Figure 6:**
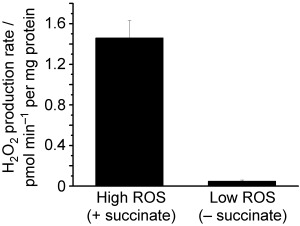
The rates of H_2_O_2_ production were monitored by using the Amplex UltraRed detection system. The high- and low-ROS conditions were created as described for Figure 5.

Building on these results, we designed an experiment to test the H_2_O_2_-dependent uncoupling that would separate the two important variables that determine uptake of MitoDNP-SUM and release of free DNP: Δ*ψ* and H_2_O_2_ production. The experimental design, which is outlined in Figure 5, allows the same Δ*ψ* to be generated at both low and high rates of H_2_O_2_ production. Complex III is inhibited by antimycin A, and Δ*ψ* is established by ATP hydrolysis. In the absence of a substrate for the ETC, there is no flow of electrons into the ETC and few ROS are produced, but when succinate is added, the stalled complex III generates a semiquinone, which reduces molecular oxygen to superoxide, and this gives H_2_O_2_ and so high ROS conditions.^23^ Thus, ROS production in the presence of succinate and antimycin A is divorced from the Δ*ψ* generated by ATP hydrolysis. This allows the Δ*ψ*-driven uptake of the MitoDNP-SUM to be the same under the high and low ROS conditions and alleviates concerns about differential accumulation of MitoDNP-SUM under the two test conditions. The difference in the rate of H_2_O_2_ production between the high and low ROS conditions was approximately 30-fold (Figure 6).

**Figure 7:**
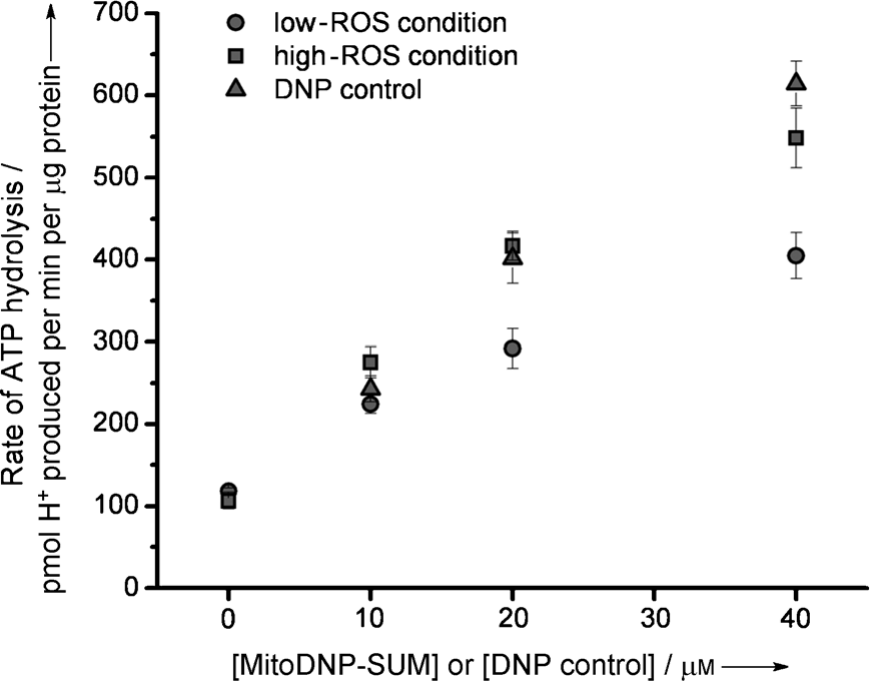
The rate of ATP hydrolysis initiated by uncoupling was measured as the rate of decline in the medium pH upon addition of MitoDNP-SUM or DNP. Experiments were performed in a Seahorse XF24 and calibrated by the addition of a known amount of acid (see the Experimental Section). Results represent means±S.E. The increased rate of ATP hydrolysis under high-ROS conditions was statistically significant under the 20 and 40 μm test conditions (*p*<0.05), as determined with a Student's unpaired *t*-test.

**Figure 8:**
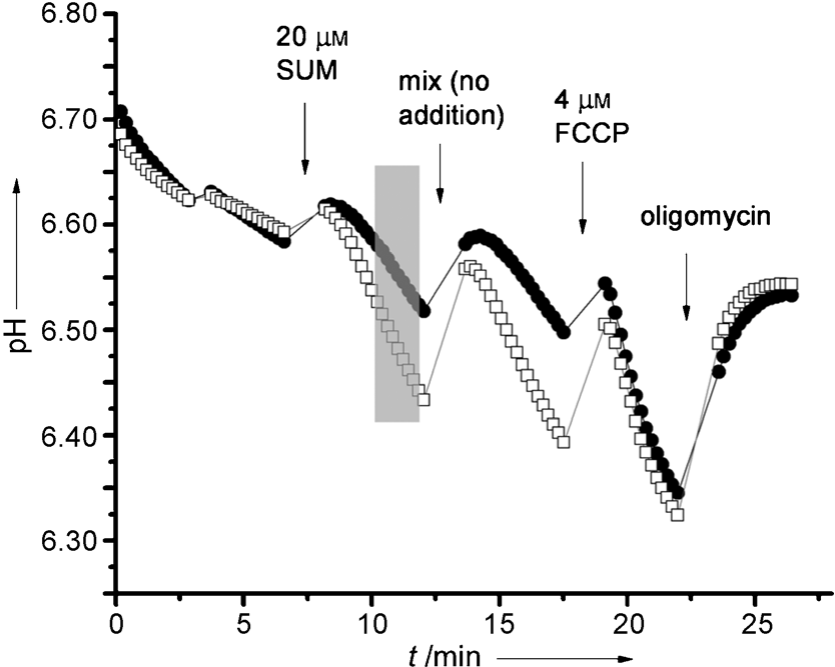
The addition of MitoDNP-SUM to mitochondria led to different rates of acidification depending on whether the experiment was conducted under low- (•) or high-ROS (□) conditions. The rate of acidification was determined by taking a linear regression of the grey shaded region on the plot. The effect of FCCP is also shown, together with the inhibition of ATP synthase by 1 μg mL^−1^ oligomycin. Typically an acid calibration addition was made at the end of multiple runs per plate, and the average pH change (∼0.15 pH units) was used to calibrate the data to pmol H^+^⋅min^−1^ per μg protein.

Hydrolysis of ATP (in this case by ATP synthase acting in reverse) generates H^+^. In the absence of an uncoupler, both ATP hydrolysis and proton translocation through ATP synthase are opposed by Δ*ψ*, but in the presence of DNP, Δ*ψ* is decreased, and ATP hydrolysis leads to net acidification of the medium outside the mitochondria. Thus, the rate of acidification reflects the amount of DNP present. The dose-dependent degree of ROS-induced uncoupling is summarised in Figure 7. There is some background uncoupling at all doses, but at the 20 and 40 μm test doses, the release of free DNP and subsequent increase in ATP hydrolysis was significantly greater under high-ROS than under low-ROS conditions (*p*<0.05), and comparable to the DNP control, thus showing that MitoDNP-SUM was successfully triggered by endogenous H_2_O_2_ to induce uncoupling in isolated mitochondria. Figure 8 illustrates this difference and the method of measurement in the example of 20 μm MitoDNP-SUM under high- and low-ROS conditions.

## Conclusions

Although there is some undesired background uncoupling induced by MitoDNP-SUM, thus making it a prototype rather than drug candidate, our study represents the first example of a mitochondria-targeted prodrug that releases its payload in direct response to mitochondria-generated ROS. MitoDNP-SUM releases a molecule known to decrease ROS production by lowering the membrane potential. Some H_2_O_2_ is produced by all active mitochondria, but high membrane potential is believed to cause disproportionately high levels of ROS, so MitoDNP-SUM represents a prototypical drug for selectively correcting the behaviour of dysfunctional mitochondria (where this dysfunction arises from high membrane potential). In a wider context, our approach provides a new way to deliver drugs specifically to the mitochondrial matrix through a combination of TPP targeting and ROS-induced uncaging.

## Experimental Section

**Synthesis**: Aldehyde **5** was prepared from 3-(4-hydroxyphenyl)propionic acid according to the literature^24^ (see the Supporting Information for details). Other reagents were obtained from commercial suppliers and used without further purification, unless otherwise stated. All reactions under an inert atmosphere were carried out in oven- or flame-dried glassware. Solutions were added via a syringe. Toluene and THF were dried, where necessary, by using a solvent drying system, Puresolv, in which solvent is pushed from its storage container under low nitrogen pressure through two stainless-steel columns containing activated alumina and copper. Acetone and triethylamine were dried by distillation from calcium sulfate and potassium hydroxide, respectively. ^1^H, ^31^P and ^13^C NMR spectra were obtained on a Bruker AVIII/500 spectrometer operating at 500, 202 and 125 MHz, respectively, or a Bruker DPX/400 spectrometer operating at 400, 162 and 100 MHz, respectively. All coupling constants are measured in Hz. Distortionless enhancement by polarisation transfer (DEPT) was used to assign the signals in the ^13^C NMR spectra as C, CH, CH_2_ or CH_3_. ESI-MS was carried out on a Thermofisher LTQ Orbitrap XL at the University of Swansea, other mass spectra were recorded on a Jeol JMS700 (MStation) spectrometer. Infra-red spectra were obtained on a Shimadzu FTIR-8400S spectrometer by using attenuated total reflectance (ATR), so that the IR spectrum of the compound (solid or liquid) could be directly detected (thin layer) without any sample preparation.

*2-(4-Iodophenyl)-4,4,5,5-tetramethyl-1,3,2-dioxaborolane (**4**):* Anhydrous THF (500 mL) was added to 1,4-diiodobenzene (**3**; 25.00 g, 75.8 mmol) and anhydrous LiCl (3.48 g, 82.1 mmol) under argon. The stirring mixture was cooled to −78 °C, and *i*PrMgCl (2.0 m in THF, 41 mL, 82 mmol) was added dropwise over 50 min. After the solution had been stirred for 3 h at −78 °C, 2-isopropoxy-4,4,5,5-tetramethyl-1,3,2-dioxaborolane (15.5 mL, 75.8 mmol) was added dropwise, and the mixture was allowed to warm slowly to RT overnight. Saturated aqueous NH_4_Cl (50 mL) was added to quench the reaction. The resulting mixture was stirred until the precipitate settled before being filtered through a pad of celite and washed through with Et_2_O. Organics were dried over MgSO_4_ and concentrated under reduced pressure. Column chromatography (SiO_2_, petroleum ether/EtOAc 9:1) yielded dioxaborolane **4** as a white solid (24.58 g, 98 %). M.p. 95–96 °C; *R*_f_=0.45 (SiO_2_, petroleum ether/EtOAc 9:1); ^1^H NMR (400 MHz, CDCl_3_): *δ*=7.72 (d, *J*=8.2 Hz, 2 H; H-2′ and H-6′), 7.51 (d, *J*=8.1 Hz, 2 H; H-3′ and H-5′), 1.33 ppm (s, 12 H; 4×Me); ^13^C NMR (100 MHz, CDCl_3_): *δ*=136.90 (CH), 136.26 (CH), 98.83 (C), 84.01 (C), 24.84 ppm (CH_3_). ^1^H and ^13^C NMR data agree with the literature.^22^

*3-(4-Methoxymethoxyphenyl)-1-[4-(4,4,5,5-tetramethyl-1,3,2-dioxaborolan-2-yl)phenyl]propan-1-ol (**6**):* Anhydrous THF (60 mL) was added to aryl iodide **4** (3.91 g, 11.8 mmol) and anhydrous LiCl (552 mg, 13.0 mmol) under argon. The stirring mixture was cooled to −78 °C, and *i*PrMgCl (2.0 m in THF, 6.5 mL, 13.0 mmol) was added dropwise over 20 min. After the mixture had been stirred for 3.5 h at −78 °C, aldehyde **5**^24^ (1.64 g, 8.4 mmol) in anhydrous THF (10 mL) was added dropwise, and the solution was allowed to warm to RT overnight. Saturated aqueous NH_4_Cl (7 mL) was added to quench the reaction. H_2_O (40 mL) was added, and the mixture was extracted with Et_2_O (3×40 mL). Combined organics were filtered through a pad of celite and washed with Et_2_O. Organics were dried over MgSO_4_ and concentrated under reduced pressure. Column chromatography (SiO_2_, petroleum ether/EtOAc 19:1 to 55:45) yielded alcohol **6** as a colourless oil (3.36 g, 93 %). *R*_f_=0.22 (SiO_2_, petroleum ether/EtOAc 4:1); ^1^H NMR (400 MHz, CDCl_3_): *δ*=7.78 (d, *J*=8.1 Hz, 2 H; H-3′′ and H-5′′), 7.32 (d, *J*=8.0 Hz, 2 H; H-2′′ and H-6′′), 7.07 (d, *J*=8.6 Hz, 2 H; H-2′ and H-6′), 6.93 (d, *J*=8.6 Hz, 2 H; H-3′ and H-5′), 5.11 (s, 2 H; OCH_2_O), 4.68–4.60 (m, 1 H; C*H*OH), 3.44 (s, 3 H; OCH_3_), 2.70–2.52 (m, 2 H; ArCH_2_), 2.33 (br s, 1 H; OH), 2.12–1.87 (m, 2 H; ArCH_2_C*H*_2_), 1.33 ppm (s, 12 H; 4×CH_3_); ^13^C NMR (100 MHz, CDCl_3_): *δ*=155.36 (C), 147.84 (C), 135.15 (C), 134.97 (CH), 129.34 (CH), 125.24 (CH), 116.25 (CH), 94.53 (CH_2_), 83.76 (C), 73.60 (CH), 55.85 (CH_3_), 40.57 (CH_2_), 31.06 (CH_2_), 24.83 ppm (CH_3_); ^11^B NMR (128 MHz, CDCl_3_): *δ*=30.54 ppm (s); IR (ATR): 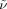
_max_=3458 (OH), 2979 (CH), 2932 (CH), 2865 (CH), 2826 (CH), 1611 (Ar), 1586 cm^−1^ (Ar); LRMS (EI^+^): *m*/*z* (%): 398 (53) [*M*]^+^, 380 (43) [*M*−H_2_O]^+^, 233 (33) [(C_6_H_12_O_2_)B(C_6_H_4_)CHOH]^+^, 230 (60), 165 [CH_3_OCH_2_O(C_6_H_4_)CH_2_CH_2_]^+^ (10), 107 (45) [HOC_6_H_4_CH_2_]^+^, 45 (100) [CH_3_OCH_2_]^+^; HRMS: 398.2260, C_23_H_31_^11^BO_5_ requires 398.2265 [*M*]^+^.

*4-{3-(2,4-Dinitrophenoxy)-3-[4-(4,4,5,5-tetramethyl-1,3,2-dioxaborolan-2-yl)phenyl]propyl}phenol (**7**):* Anhydrous acetone (0.2 mL) was added to a stirring solution of alcohol **6** (500 mg, 1.26 mmol), 1-fluoro-2,4-dinitrobenzene (470 μL, 3.77 mmol) and anhydrous triethylamine (525 μL, 3.77 mmol) under argon. The resulting solution was stirred at 40 °C for 72 h, then concentrated under reduced pressure. EtOAc (12 mL) was added, and the solution was washed with saturated aqueous NaHCO_3_ (6 mL) and H_2_O (2×6 mL). Organics were dried over MgSO_4_ and concentrated under reduced pressure. CH_2_Cl_2_ (16 mL) and MeOH (16 mL) were added followed by concentrated HCl (4 mL) dropwise. The resulting solution was stirred at 32 °C for 3.5 h. Pinacol (35 mg, 296 μmol) and 4 Å molecular sieves were added, and the mixture was stirred at RT for a further 1 h. The solution was filtered, and sieves were washed with CH_2_Cl_2_ (10 mL). Organics were poured into aqueous HCl (1 m, 12 mL) and separated before the mixture was extracted with CH_2_Cl_2_ (3×10 mL). Combined organics were dried over MgSO_4_ and concentrated under reduced pressure. Column chromatography (SiO_2_, petroleum ether/EtOAc 9:1 to 2:1) yielded phenol **7** as a yellow oil (417 mg, 64 %). *R*_f_ (SiO_2_, petroleum ether/EtOAc 7:3)=0.33; ^1^H NMR (500 MHz, CDCl_3_): *δ*=8.70 (d, *J*=2.8 Hz, 1 H; H-3′′), 8.14 (dd, *J*=9.3, 2.8 Hz, 1 H; H-5′′), 7.80 (d, *J*=7.9 Hz, 2 H; H-3′′′ and H-5′′′), 7.31 (d, *J*=8.0 Hz, 2 H; H-2′′′ and H-6′′′), 7.02 (d, *J*=8.4 Hz, 2 H; H-3 and H-5), 6.85 (d, *J*=9.4 Hz, 1 H; H-6′′), 6.74 (d, *J*=8.4 Hz, 2 H; H-2 and H-6), 5.25 (dd, *J*=8.4 and 4.5 Hz, 1 H; C*H*ODNB), 4.81 (br s, 1 H; OH), 2.77 (t, *J*=7.5 Hz, 2 H; ArCH_2_), 2.46–2.36 (m, 1 H; ArCH_2_C*H*^A^H^B^), 2.21–2.11 (m, 1 H; ArCH_2_CH^A^*H*^B^), 1.33 (s, 6 H; 2×Me), 1.32 ppm (s, 6 H; 2×Me); ^13^C NMR (126 MHz, CDCl_3_): *δ*=155.71 (C), 153.96 (C), 141.82 (C), 139.92 (C), 139.40 (C), 135.61 (CH), 132.59 (C), 129.60 (CH), 128.56 (CH), 125.10 (CH), 121.69 (CH), 116.00 (CH), 115.46 (CH), 84.06 (C), 82.12 (CH), 32.92 (CH_2_), 30.48 (CH_2_), 24.87 (CH_3_), 24.82 ppm (CH_3_); LRMS (EI^+^): *m*/*z* (%): 520 (7) [*M*]^+^, 336 (7) [*M*−DNP]^+^, 184 (10) [DNP]^+^, 107 (100) [HOArCH_2_]^+^, 91 (16); HRMS: 520.2018 and 519.2049, C_27_H_29_^11^BN_2_O_8_ requires 520.2017 [*M*]^+^ and C_27_H_29_^10^BN_2_O_8_ requires 519.2053 [*M*]^+^.

*[4-(Bromomethyl)benzyl]triphenylphosphonium bromide (**8**):* A solution of triphenylphosphine (100 mg, 0.38 mmol) in anhydrous toluene (1.25 mL) was added dropwise to a stirring solution of α,α′-*para*-dibromoxylene (604 mg, 2.3 mmol) in anhydrous toluene (2.5 mL) at 95 °C under argon, and the resulting solution was stirred for 1 h. A further solution of triphenylphosphine (100 mg, 0.38 mmol) in anhydrous toluene (2.5 mL) was added dropwise, and the resulting mixture was stirred for 5 h at 95 °C under argon. The hot mixture was filtered, and the precipitate was washed with hot toluene and then Et_2_O. The solid was dried under reduced pressure to yield phosphonium bromide **8** as a white amorphous solid (391 mg, 98 %). M.p.>220 °C (decomp.); ^1^H NMR (400 MHz, CD_3_CN): *δ*=7.91–7.84 (m, 3 H; 3×*p*-H PPh_3_), 7.72–7.64 (m, 6 H; 6×*o*-H PPh_3_), 7.61–7.51 (m, 6 H; 6×*m*-H PPh_3_), 7.27 (d, *J*=7.7 Hz, 2 H; H-3 and H-5), 6.94 (dd, *J*=7.9, 1.8 Hz, 2 H; H-2 and H-6), 4.68 (d, *J*=14.3 Hz, 2 H; CH_2_P), 4.52 ppm (s, 2 H; CH_2_Br); ^13^C NMR (100 MHz, CD_3_CN): *δ*=138.89 (d, *J*=4.6 Hz, C), 134.95 (d, *J*=2.9 Hz, CH), 133.89 (d, *J*=9.8 Hz, CH), 131.01 (d, *J*=5.4 Hz, CH), 129.77 (d, *J*=12.6 Hz, CH), 129.29 (d, *J*=3.0 Hz, CH), 127.21 (d, *J*=7.8 Hz, C), 117.06 (d, *J*=86.0 Hz, C), 32.36 (s, CH_2_), 29.16 ppm (d, *J*=48.3 Hz, CH_2_); {^1^H}^31^P NMR (162 MHz, CD_3_CN): *δ*=22.71 ppm (s); IR (ATR): 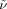
_max_=3054 (CH), 3010 (CH), 2990 (CH), 2965 (CH), 2887 (CH), 2850 (CH), 2779 (CH), 1604 (Ar), 1588 (Ar), 1572 cm^−1^ (Ar); LRMS (ESI^+^): 447 [cation (^81^Br), 100 %], 445 [cation (^79^Br), 93]; HRMS: 447.0683 and 445.0707, C_26_H_26_^81^BrP requires cation, 447.0695 and C_26_H_26_^79^BrP requires cation, 445.0715; LRMS (ESI^−^): 81 (^81^Br^−^, 99 %) and 79 (^79^Br^−^, 100).

*[(4-{4-(3-{2,4-Dinitrophenoxy}-3-{4-(4,4,5,5-tetramethyl-1,3,2-dioxaborolan-2-yl)phenyl}prop-1-yl)phenoxymethyl}phenyl)methyl]triphenylphosphonium bromide (MitoDNP-SUM):* Cs_2_CO_3_ (178 mg, 0.55 mmol) was added to phenol **5** (178 mg, 0.34 mmol) in anhydrous MeCN (1.2 mL), and the mixture was stirred under argon for 5 min. Benzylic bromide **8** (180 mg, 0.34 mmol) in anhydrous CH_2_Cl_2_ (0.4 mL) was added. The resulting solution was stirred at RT for 10.5 h under argon before being concentrated under reduced pressure. H_2_O (5 mL) was added, and the resulting solution was extracted with CHCl_3_ (3×5 mL). Combined organics were dried over MgSO_4_ and concentrated under reduced pressure. The solid was dissolved in CH_2_Cl_2_ and precipitated by being added dropwise to stirring Et_2_O. The liquor was pipetted off, and the solid was washed with Et_2_O. Column chromatography of the residual solid (SiO_2_, CH_2_Cl_2_/EtOH 9:1) yielded MitoDNP-SUM as a yellow amorphous solid (192 mg, 58 %). *R*_f_ (SiO_2_, CH_2_Cl_2_/EtOH 9:1)=0.20; ^1^H NMR (500 MHz, CDCl_3_): *δ*=8.69 (d, *J*=2.8 Hz, 1 H; H-3′′′), 8.14 (dd, *J*=9.3 and 2.8 Hz, 1 H; H-5′′′), 7.82–7.72 (m, 11 H; 6×*o*-H PPh_3_, 3×*p*-H PPh_3_, H-3 and H-5), 7.66–7.59 (m, 6 H; 6×*m*-H PPh_3_), 7.31 (d, *J*=8.1 Hz, 2 H; H-3′′′′ and H-5′′′′), 7.19 (d, *J*=8.0 Hz, 2 H; H-2′′′′ and H-6′′′′), 7.13 (dd, *J*=8.3, 2.8 Hz, 2 H; H-2 and H-6), 7.05 (d, *J*=8.7 Hz, 2 H; H-3′ and H-5′), 6.86 (d, *J*=9.4 Hz, 1 H; H-6′′′), 6.81 (d, *J*=8.7 Hz, 2 H; H-2′ and H-6′), 5.50–5.42 (m, 2 H; CH_2_P), 5.26 (dd, *J*=8.4, 4.5 Hz, 1 H; H-3′′), 4.96 (d, *J*=2.0 Hz, 2 H; CH_2_O), 2.77 (t, *J*=7.5 Hz, 2 H; H-1′′), 2.45–2.36 (m, 1 H; H^A^-2′′), 2.21–2.12 (m, 1 H; H^B^-2′′), 1.324 (s, 6 H; 2×Me), 1.319 ppm (s, 6 H; 2×Me);^13^C NMR (126 MHz, CDCl_3_): *δ*=156.86 (C), 155.73 (C), 141.79 (C), 139.77 (C), 139.22 (C), 137.53 (d, *J*=4.2 Hz, C), 135.54 (CH), 135.07 (d, *J*=2.4 Hz, CH), 134.32 (d, *J*=9.8 Hz, CH), 133.05 (C), 131.63 (d, *J*=5.6 Hz, CH), 130.18 (d, *J*=12.7 Hz, CH), 129.41 (CH), 128.72 (CH), 127.66 (d, *J*=3.0 Hz, CH), 126.62 (d, *J*=8.6 Hz, C), 125.14 (CH), 121.59 (CH), 117.61 (d, *J*=85.6 Hz, C), 116.32 (CH), 114.95 (CH), 83.99 (C), 82.10 (CH), 69.29 (CH_2_), 39.89 (CH_2_), 30.49 (d, *J*=47.3 Hz, CH_2_), 30.46 (CH_2_), 24.86 (CH_3_), 24.81 ppm (CH_3_); {^1^H}^31^P NMR (162 MHz, CDCl_3_): *δ*=23.00 ppm (s); IR (ATR): 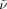
_max_=3059 (CH), 3051 (CH), 2978 (CH), 2918 (CH), 2868 (CH), 2853 (CH), 1605 (Ar), 1524 (Ar), 1510 (Ar); LRMS (ESI^+^): 885 (cation, 100 %); HRMS (ESI^+^): 885.3478 and 884.3517. C_53_H_51_^11^BN_2_O_8_P requires cation, 885.3479 and C_53_H_51_^10^BN_2_O_8_P requires cation, 884.3507; LRMS (ESI^−^): 81 (^81^Br^−^, 98 %) and 79 (^79^Br^−^, 100).

**Biology**

*Animals, reagents and mitochondrial preparation:* Female Wister rats (Harlan Laboratories), age 5–8 weeks, were fed chow ad libitum with free access to water. Skeletal muscle mitochondria were isolated at 4 °C in Chappell–Perry buffer (CP1; 100 mm KCl, 50 mm Tris, 2 mm ethylene glycol tetraacetic acid (EGTA), pH 7.1 at 25 °C) by standard procedures.^25^ The animal protocol was approved by the Buck Institute Animal Care and Use Committee, in accordance with IACUC standards. All reagents were purchased from Sigma– Aldrich except Amplex UltraRed, which was purchased from Invitrogen.

*Superoxide and H_2_O_2_ production by isolated mitochondria:* The combined superoxide and H_2_O_2_ production rates were measured as H_2_O_2_ production rate after dismutation of superoxide by endogenous superoxide dismutase. H_2_O_2_ production rates were determined by using the Amplex UltraRed detection system in a Cary Varian spectrofluorometer at the wavelength couple *λ*_ex_=560 nm, *λ*_em_=590 nm. Horseradish peroxidase was added to catalyse reaction of extramitochondrial H_2_O_2_ with Amplex UltraRed to form a fluorescent resorufin product. Mitochondria (0.3 mg protein per mL) were incubated at 37 °C in standard medium containing KCl (120 mm), 4-(2-hydroxyethyl)-1-piperazineethanesulfonic acid (HEPES, 5 mm), EGTA (1 mm, pH 7.2 at 20 °C) and bovine serum albumin (0.3 %, *w*/*v*). All assays contained Amplex UltraRed (50 μm) and horseradish peroxidase (5 units per mL). Fluorescence emission data were calibrated from H_2_O_2_ standard curves obtained under identical conditions.

*Methyltriphenylphosphonium (TPMP) electrode detection of MitoDNP-SUM uptake:* TPMP electrodes were prepared exactly as described in the literature.^25^ Skeletal muscle mitochondria were incubated under similar conditions as for H_2_O_2_ production measurements at 37 °C in KHE medium. Succinate oxidation generated a Δ*p* of ∼180 mV, and ATP hydrolysis routinely generated a Δ*p* of ∼140 mV; these were measured by using TPMP distribution in the presence of nigericin (100 nm) and were similar to values in the literature.^26^ The uptake of MitoDNP-SUM was monitored after addition of the compound (2×3 μm). Higher concentrations and more additions could not be assessed because high amounts of MitoDNP-SUM interfered with the TPMP electrode.

*MitoDNP-SUM ROS-dependent uncoupling:* All uncoupling assays were performed by using a Seahorse XF24 Extracellular Flux Analyzer (Seahorse Bioscience). The instrument assays O_2_ and H^+^ levels by using fluorescent probes on a sensor cartridge that lowers to within 200 μm of the well bottom during a measurement cycle, thereby creating a trapped volume (7 μL) in which the measurement is made. Isolated skeletal muscle mitochondria (5 μg per well) were spun down onto 24-well plates exactly as described in the literature.^27^ The assay was performed in standard KHE medium containing ATP (5 mm), antimycin A (2 μm), and the presence or absence of succinate created the high- or low-ROS conditions, as described above. The medium was maintained at pH 6.8 and 37 °C. The XF24 cartridge has four reagent delivery ports per well for injecting compounds into the wells during an assay. MitoDNP-SUM was injected at the first port, and ATP hydrolysis was monitored as a drop in pH over time (3 min). In multiple wells on the plate, HCl (37.5 nmol) was added at the end of the experiment to acidify the solution by about 0.15 pH units as an acid calibration point, and data were converted to pmol H^+^⋅min^−1^ per μg protein.

## Supplementary material

As a service to our authors and readers, this journal provides supporting information supplied by the authors. Such materials are peer reviewed and may be re-organized for online delivery, but are not copy-edited or typeset. Technical support issues arising from supporting information (other than missing files) should be addressed to the authors.
