# Supplementary material for: A Prototypical Small-Molecule Modulator Uncouples Mitochondria in Response to Endogenous Hydrogen Peroxide Production
Source: Chembiochem. 2013 May 2;14(8):993–1000. doi: 10.1002/cbic.201300115 (PMC3743171; doi:10.1002/cbic.201300115)
Supplement: Supplementary file 1 [file cbic0014-0993-SD1.pdf]

CHEMBIOCHEM

Supporting Information

© Copyright Wiley-VCH Verlag GmbH & Co. KGaA, 69451 Weinheim, 2013

A Prototypical Small-Molecule Modulator Uncouples Mitochondria in Response to Endogenous Hydrogen Peroxide Production

Stephen J. McQuaker,[a] Casey L. Quinlan,[b] Stuart T. Caldwell,[a] Martin D. Brand,[b] and Richard C. Hartley*[a]

cbic_201300115_sm_miscellaneous_information.pdf

Supplementary material:

A prototypical small molecule modulator uncouples mitochondria in response to endogenous hydrogen peroxide production.

Stephen J. McQuaker,[a] Casey L. Quinlan,[b] Stuart T. Caldwell,[a] Martin D. Brand,[b] and Richard C. Hartley.[a]*
[a]Centre for the Chemical Research of Ageing, WestCHEM School of Chemistry, University of Glasgow, Glasgow, G12 8QQ, UK
[b]Buck Institute for Research on Aging, 8001 Redwood Boulevard, Novato, California 94945, USA.

Synthesis of aldehyde 5 and phosphonium salt 8.

Supplementary Scheme 1 (a) The synthesis of aldehyde **5** from carboxylic acid **9**: the acid **9** was converted into ester **10** and the phenol protected as MOM ether **11**; reduction to the alcohol **12**, followed by Swern oxidation then gave aldehyde **5**. (b) The synthesis of phosphonium salt **8** from α, α'-*para*-dibromoxylene **13**.

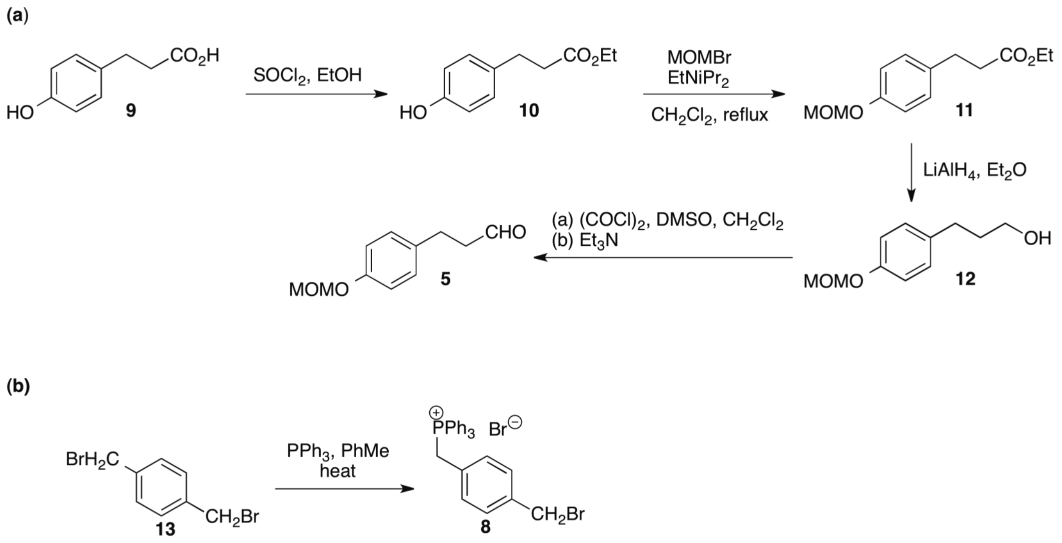

<u>Ethyl 3-(4'-hydroxyphenyl)propionate 10</u>

Thionyl chloride (10.54 mL, 144.4 mmol) was added dropwise to a stirring solution of 3-(4'-hydroxyphenyl)propionic acid **9** (8.00 g, 48.1 mmol) in EtOH (150 mL). The solution was stirred at reflux for 4.5 h. After cooling to RT, the solution was concentrated under reduced pressure to give a crude orange oil. Column chromatography [SiO_2, petroleum ether-EtOAc (9:1) to (3:2)] yielded ethyl ester **10** as an oil that solidified on standing (9.30 g, 99%). R_f [SiO_2, petroleum ether-EtOAc (7:3)]: 0.47. Mp: 42-43 °C. ν_{max} (ATR): 3364 (OH), 2998 (CH), 2981 (CH), 2962 (CH), 2942 (CH), 2904 (CH), 2870 (CH), 1701 (C=O), 1611 (Ar), 1596 (Ar) cm^{-1}. δ_H (400 MHz, $CDCl_3$): 7.04 (2H, d, J = 8.6 Hz, H-2' and H-6'), 6.74 (2H, d, J = 8.6 Hz, H-3' and H-5'), 6.02 (1H, broad s, OH), 4.13 (2H, q, J = 7.1 Hz, CH_2CH_3), 2.87 (2H, t, J = 7.9 Hz, 2 × H-3), 2.59 (2H, t, J = 7.8 Hz, 2 × H-2), 1.23 (3H, t, J = 7.2 Hz, CH_3). δ_C (100 MHz, $CDCl_3$): 173.75 (C), 154.24 (C), 132.23 (C), 129.38 (CH), 115.33 (CH), 60.72 (CH_2), 36.32 (CH_2), 30.10 (CH_2), 14.15 (CH_3). LRMS (EI^+): 194 ($M^{+\bullet}$, 33%), 149 ($M^{+\bullet}$ – $EtO^{\bullet}$, 9), 120 ($M^{+\bullet}$ – EtOH and – CO_2, 78), 107 ($HOC_6H_4CH_2^+$, 100), 83 (68). HRMS: 194.0945. $C_{11}H_{14}O_3$ requires $M^{+\bullet}$, 194.0943. ^1H NMR data agree with literature.[S1]

Ethyl 3-(4'-methoxymethoxyphenyl)propionate 11

A stirring solution of ethyl ester **10** (27.00 g, 139 mmol) and *N*, *N*-diisopropylethylamine (31.5 mL, 181 mmol) in anhydrous DCM (400 mL) was degassed with argon for 30 min. Bromomethyl methyl ether (16.5 mL, 90%, 181 mmol) was added dropwise and the resulting solution stirred at reflux overnight under argon. After cooling to RT, the mixture was washed with aqueous HCl (1 M, 150 mL), H_2O (2 × 150 mL), dried over $MgSO_4$ and concentrated under reduced pressure. Column chromatography [SiO_2, petroleum ether-EtOAc (19:1) to (65:35)] yielded ethyl ester **11** as an oil (31.12 g, 94%). R_f [SiO_2, petroleum ether-EtOAc (4:1)]: 0.49. ν_{max} (ATR): 2982 (CH), 2955 (CH), 2936 (CH), 2826 (CH), 1732 (C=O), 1613 (Ar), 1586 (Ar) cm^{-1}. δ_H (400 MHz, $CDCl_3$): 7.11 (2H, d, *J* = 8.7 Hz, H-2' and H-6'), 6.94 (2H, d, *J* = 8.7 Hz, H-3' and H-5'), 5.13 (2H, s, OCH_2O), 4.11 (2H, q, *J* = 7.1 Hz, CH_2CH_3), 3.45 (3H, s, OMe), 2.88 (2H, t, *J* = 7.8 Hz, 2 × H-3), 2.57 (2H, t, *J* = 7.9 Hz, 2 × H-2), 1.22 (3H, t, *J* = 7.2 Hz, CH_2CH_3). δ_C (100 MHz, $CDCl_3$): 172.87 (C), 155.72 (C), 133.98 (C), 129.29 (CH), 116.30 (CH), 94.49 (CH_2), 60.33 (CH_2), 55.85 (CH_3), 36.15 (CH_2), 30.17 (CH_2), 14.21 (CH_3). LRMS (EI^+): 238 ($M^{+\bullet}$, 51%), 45 ($CH_3OCH_2^+$, 100). HRMS: 238.1209. $C_{13}H_{18}O_4$ requires $M^{+\bullet}$, 238.1205. Compound reported in literature without characterisation data.[S2]

3-(4'-Methoxymethoxyphenyl)propan-1-ol 12

A stirring solution of ethyl ester **11** (8.73 g, 36.6 mmol) in anhydrous Et_2O (140 mL) was cooled to 0 °C under argon and $LiAlH_4$ (4.17 g, 109.8 mmol) was added portionwise. The solution was stirred at 0 °C under argon for 45 min before being allowed to warm to RT overnight. H_2O (25 mL) was added dropwise to quench. H_2O (20 mL) and Et_2O (100 mL) were added to dilute and the mixture stirred until the precipitate settled. The organic layer was filtered through a pad of celite and washed through with Et_2O. Organics were dried over $MgSO_4$ and concentrated under reduced pressure to yield alcohol **12** as an oil (6.05 g, 84%). ν_{max} (ATR): 3347 (OH), 2994 (CH), 2937 (CH), 2902 (CH), 2861 (CH), 2827 (CH), 2785 (CH), 1612 (Ar), 1585 (Ar) cm^{-1}. δ_H (400 MHz, $CDCl_3$): 7.08 (2H, d, *J* = 8.7 Hz, H-2' and H-6'), 6.94 (2H, d, *J* = 8.7 Hz, H-3' and H-5'), 5.11 (2H, s, OCH_2O), 3.59 (2H, broad t, *J* = 5.9 Hz, 2 × H-1), 3.44 (3H, s, OMe), 3.00 (1H, broad s, OH), 2.61 (2H, t, *J* = 7.7 Hz, 2 × H-3), 1.86-1.77 (2H, m, 2 × H-2). δ_C (100 MHz, $CDCl_3$): 155.31 (C), 135.36 (C), 129.33 (CH), 116.23 (CH), 94.50 (CH_2), 61.81 (CH_2), 55.84 (CH_3), 34.34 (CH_2), 31.17 (CH_2). LRMS (CI^+): 197 [$(M + H)^+$, 46%], 165 [$(M + H)^+$ – MeOH, 100]. HRMS: 197.1179. $C_{11}H_{17}O_3$ requires $(M + H)^+$, 197.1178. ^1H and ^{13}C NMR are in agreement with literature, excluding the misreported ^{13}C NMR peak at 192.25 ppm (corresponds to peak the CH at 129.33 ppm above).[24]

3-(4'-Methoxymethoxyphenyl)propionaldehyde 5

Oxalyl chloride (1.9 mL, 21.6 mmol) was added to anhydrous DCM (70 mL) at –78 °C under argon and stirred for 5 min. Anhydrous DMSO (2.7 mL, 38.0 mmol) was added and the resulting solution stirred at –78 °C for 30 min. A solution of alcohol **12** (3.00 g, 15.3 mmol) in anhydrous DCM (20 mL) was added slowly. After stirring for 30 min, anhydrous triethylamine (10.65 mL, 76.4 mmol) was added. After stirring for a further 30 min at –78 °C, the solution was allowed to warm to RT and stir for a further 2.5 h. The reaction was concentrated under reduced pressure. H_2O (50 mL) was added and extractions were made with DCM (3 × 30 mL). Combined organics were dried over $MgSO_4$ and concentrated under reduced pressure. Column chromatography [SiO_2, petroleum ether-EtOAc (9:1) to (7:3)] yielded aldehyde **5** as an oil (2.97 g, 94%). R_f [SiO_2, petroleum ether-EtOAc (4:1)]: 0.44. ν_{max} (ATR): 2996 (CH), 2957 (CH), 2933 (CH), 2898 (CH), 2848 (CH), 2826 (CH), 2792 (CH), 1722 (C=O), 1611 (Ar), 1585 (Ar) cm^{-1}. δ_H (400 MHz, $CDCl_3$): 9.78 (1H, t, *J* = 1.4 Hz, H-1), 7.10 (2H, d, *J* = 8.7 Hz, H-2' and H-6'), 6.96 (2H, d, *J* = 8.6 Hz, H-3' and H-5'), 5.13 (2H, s, OCH_2O), 3.45 (3H, s, OMe), 2.89 (2H, t, 2 × H-3, *J* = 7.5 Hz), 2.73 (2H, broad t, 2 × H-2, *J* = 7.5 Hz. δ_C (100 MHz, $CDCl_3$): 201.60 (CH), 155.73 (C), 133.72 (C), 129.26 (CH), 116.42 (CH), 94.48 (CH_2), 55.84 (CH_3), 45.39 (CH_2), 27.27 (CH_2). ^1H and ^{13}C NMR data agree with literature.[24]

[4-(Bromomethyl)benzyl]triphenylphosphonium bromide 8

A solution of triphenylphosphine (100 mg, 0.38 mmol) in anhydrous toluene (1.25 mL) was added dropwise to a stirring solution of α, α'-*para*-dibromoxylene **13** (604 mg, 2.3 mmol) in anhydrous toluene (2.5 mL) at 95 °C under argon and stirred for 1 h. A further solution of triphenylphosphine (100 mg, 0.38 mmol) in anhydrous toluene (2.5 mL) was added dropwise and the resulting mixture was stirred for 5 h at 95 °C under argon. The hot mixture was filtered and the precipitate washed with hot toluene and then Et_2O. The solid was dried under reduced pressure to yield phosphonium bromide **8** as an amorphous solid (391 mg, 98%). Mp: >220 °C (Decomp.). ν_{max} (ATR): 3054 (CH), 3010 (CH), 2990 (CH), 2965 (CH), 2887 (CH), 2850 (CH), 2779 (CH), 1604 (Ar), 1588 (Ar), 1572 (Ar). δ_H (400 MHz, CD_3CN): 7.91-7.84 (3H, m, 3 × *p*-H PPh_3), 7.72-7.64 (6H, m, 6 × *o*-H PPh_3), 7.61-7.51 (6H, m, 6 × *m*-H PPh_3), 7.27 (2H, d, *J* = 7.7 Hz, H-3 and H-5), 6.94 (2H, dd, *J* = 7.9 and 1.8 Hz, H-2 and H-6), 4.68 (2H, d, *J* = 14.3 Hz, CH_2P), 4.52 (2H, s, CH_2Br). δ_C (100 MHz, CD_3CN): 138.89 (d, *J* = 4.6 Hz, C), 134.95 (d, *J* = 2.9 Hz, CH), 133.89 (d, *J* = 9.8 Hz, CH), 131.01 (d, *J* = 5.4 Hz, CH), 129.77 (d, *J* = 12.6 Hz, CH), 129.29 (d, *J* = 3.0 Hz, CH), 127.21 (d, *J* = 7.8 Hz, C), 117.06 (d, *J* = 86.0 Hz, C), 32.36 (s, CH_2), 29.16 (d, *J* = 48.3 Hz, CH_2). d_P {^1H} (162 MHz, CD_3CN): 22.71 (s). LRMS (ESI^+): 447 [cation (^{81}Br), 100%], 445 [cation (^{79}Br), 93]. HRMS: 447.0683 and 445.0707. $C_{26}H_{26}{}^{81}BrP$ requires cation, 447.0695 and $C_{26}H_{26}{}^{79}BrP$ requires cation, 445.0715. LRMS (ESI^-): 81 ($^{81}Br^-$, 99%) and 79 ($^{79}Br^-$, 100).

References

S1. P. Sauerberg, G. S. Olsen, L. Jeppesen, J. P. Mogensen, I. Pettersson, C. B. Jeppesen, J. R. Daugaard, E. D. Galsgaard, L. Ynddal, J. Fleckner, V. Panajotova, Z. Polivka, P. Pihera, M. Havranek, E. M. Wulff, *J. Med. Chem.* **2007**, *50*, 1495-1503.

S2. P. Molina, P. M. Fresneda, M. A. Sanz, *J. Org. Chem.* **1999**, *64*, 2540-2544.
